# The effect of reduced scan time on response assessment FDG-PET/CT imaging using Deauville score in patients with lymphoma

**DOI:** 10.1186/s41824-021-00096-0

**Published:** 2021-01-26

**Authors:** Charlotte Hornnes, Annika Loft, Liselotte Højgaard, Flemming Littrup Andersen

**Affiliations:** Department of Clinical Physiology, Nuclear Medicine and PET, Rigshospitalet, University of Copenhagen, Blegdamsvej 9, DK-2100 Copenhagen, Denmark

**Keywords:** Lymphoma, PET/CT, Deauville score, Acquisition time, Image quality, Cost savings

## Abstract

**Purpose:**

[^18^F]Fluoro-deoxy-glucose positron emission tomography/computed tomography (FDG-PET/CT) is used for response assessment during therapy in Hodgkin lymphoma (HL) and non-Hodgkin lymphoma (NHL). Clinicians report the scans visually using Deauville criteria. Improved performance in modern PET/CT scanners could allow for a reduction in scan time without compromising diagnostic image quality. Additionally, patient throughput can be increased with increasing cost-effectiveness. We investigated the effects of reducing scan time of response assessment FDG-PET/CT in HL and NHL patients on Deauville score (DS) and image quality.

**Methods:**

Twenty patients diagnosed with HL/NHL referred to a response assessment FDG-PET/CT were included. PET scans were performed in list-mode with an acquisition time of 120 s per bed position(s/bp). From PET list-mode data images with full acquisition time of 120 s/bp and shorter acquisition times (90, 60, 45, and 30 s/bp) were reconstructed. All images were assessed by two specialists and assigned a DS. We estimated the possible savings when reducing scan time using a simplified model based on assumed values/costs for our hospital.

**Results:**

There were no significant changes in the visually assessed DS when reducing scan time to 90 s/bp, 60 s/bp, 45 s/bp, and 30 s/bp. Image quality of 90 s/bp images were rated equal to 120 s/bp images. Coefficient of variance values for 120 s/bp and 90 s/bp images was significantly < 15%. The estimated annual savings to the hospital when reducing scan time was 8000-16,000 €/scanner.

**Conclusion:**

Acquisition time can be reduced to 90 s/bp in response assessment FDG-PET/CT without compromising Deauville score or image quality. Reducing acquisition time can reduce costs to the clinic.

## Introduction

Malignant lymphoma is the most common hematological malignancy in the world (Bray et al., 2018). The two main types of lymphoma are Hodgkin lymphoma (HL) and non-Hodgkin lymphoma (NHL). In 2018, 79,990 new cases of HL and 509,590 new cases of NHL were reported worldwide (Bray et al., 2018). The incidence of NHL increases with age whereas HL mainly affects young adults (Smith et al., 2015).

In recent years, [^18^F]fluoro-deoxy-glucose positron emission tomography/computed tomography (FDG-PET/CT) has become the standard imaging procedure for initial staging and response assessment both during therapy and end of therapy in HL and FDG-avid NHL (Cheson et al., 2014). PET detects areas with an increased FDG uptake in the body, identifying cells with a high glucose metabolism as is the case in most malignant cells (Boellaard et al., 2015). FDG-avid areas are located anatomically by simultaneously performing a CT scan. Both HL and NHL are typically very FDG-avid (Weiler-Sagie et al., 2010). FDG-PET/CT has improved diagnostic accuracy in comparison to CT for staging in HL and NHL, with increased sensitivity for extranodal sites, and is now recommended for staging (Barrington et al., 2014). Response assessment of lymphoma patients with an interim FDG-PET/CT is performed during treatment to ensure the effectiveness of treatment. Interim PET has proven to be a strong prognostic indicator in HL and NHL (Gallamini et al., 2007; Mikhaeel et al., 2005; Itti et al., 2013).

In 2009, the First International Workshop on interim PET scan in lymphoma proposed the Deauville criteria for reporting interim PET scans (Meignan et al., 2009). The criteria use a five-point scale based on a visual interpretation of FDG uptake, comparing FDG uptake in sites of (initial) lymphoma with FDG uptake in two reference organs—the liver and the mediastinal blood pool. The scale has been validated for use at interim PET and has shown good inter-observer reproducibility in HL and NHL (Itti et al., 2013; Biggi et al., 2013; Barrington et al., 2010).

PET/CT is a time-consuming examination. It is desirable to reduce scan time to improve patient comfort and compliance and possibly increase patient throughput and cost-effectiveness of the examination. Another important factor is that PET and CT utilize ionizing radiation and one risk attached to performing PET/CT is therefore inducing de novo cancer (Huang et al., 2009). Even though the risk is estimated to be very little, patients with HL and NHL undergo repetitive PET/CT imaging during staging and treatment causing radiation exposure to the patient and to the scan performing staff. Therefore, according to the ALARA principle, any possibility of reducing radiation must be addressed.

Novel PET/CT scanners have an improved sensitivity compared to older scanners (van der Vos et al., 2017). This is due to several factors including a longer gantry, which increases the number of detected counts and a significantly improved time-of-flight (TOF) resolution in new scanners (Conti & Bendriem, 2019). Thus, a reduction in scan time and tracer dose might be feasible for PET/CT examinations performed on new scanners developed since the onset of the Deauville criteria, without compromising the clinical diagnostic quality. In addition, reducing the acquisition time per bed position in FDG-PET/CT examinations could increase the patient throughput, reduce costs to the personnel operating the scanner and thereby increase the cost-effectiveness.

The aim of this study is to investigate the effect of reducing scan time of response assessment PET/CT imaging performed on newly diagnosed patients with HL and NHL on Deauville score (DS) and image quality. Furthermore, we want to quantify the possible savings in healthcare costs from the eventual reduction of scan time.

## Materials and methods

### Study design and patients

The study was designed as a prospective study including 23 consecutive patients with newly diagnosed malignant lymphoma, who had a PET/CT for staging performed, and were referred to a response assessment FDG-PET/CT from the Department of Hematology, Rigshospitalet, University of Copenhagen, Denmark, from October 2019 until May 2020. Exclusion criteria were patients with non-FDG-avid lymphomas on baseline PET/CT and age younger than 18 years. Three patients were excluded due to complete response with no visible tumor on the interim PET, resulting in a final cohort of 20 patients.

All patients underwent an initial PET/CT scan at diagnosis (baseline PET) and a subsequent response assessment PET/CT (after 1-4 cycles of chemotherapy). The following clinical data were obtained from all patients: sex, age, diagnosis, date of baseline PET, and FDG-avidity on baseline PET.

According to the Danish National Committee on Health Research, ethics approval was not required in this study as it qualifies as a quality assurance study (Journal-no: H-19075323). Patient data were fully anonymized before data analysis, and the GDPR rules are thereby fulfilled.

### FDG-PET/CT imaging

The PET/CT scanning was performed on a Siemens Biograph Vision 600 system (Siemens Healthineers, Erlangen) in the Department of Clinical Physiology, Nuclear Medicine and PET, Rigshospitalet, University of Copenhagen, Denmark.

The scans were acquired according to EANM procedure guidelines for tumor imaging (Boellaard et al., 2015). Patients fasted for a minimum of 4 h prior to intravenous injection of 4 MBq ^18^F-FDG/kg body weight, slightly above the recommended minimum dose of 3.5 MBq ^18^F-FDG/kg body weight to match institutional standard. Scans were performed approximately 60 min after injection and with a bed overlap of 49.7%.

Low-dose CTs were typically acquired at 120 kVp adjusted using CareKV and using 40 mAs. One patient had a diagnostic CT performed due to clinical requirement. Following CT, a PET scan was performed with an acquisition time of 120 s per bed position (s/bp) and acquired in list mode. Scans were covering from the base of the skull to the proximal thigh. CT data was used for attenuation correction of the PET data. Both PET and CT images were reconstructed with a slice thickness of 2 mm.

The PET list-mode data was used to reconstruct images with a full acquisition time of 120 s/bp. Subsequently, list-mode data was randomly subsampled to simulate images acquired with a reduced acquisition time of 90 s/bp, 60 s/bp, 45 s/bp, and 30 s/bp. All images were reconstructed into 440 × 440 matrices with 3D ordered Poisson subset expectation maximization (3D-OP-OSEM) using 4 iterations, 5 subsets, and TOF; no point spread function (PSF) modeling was used. Data were post-filtered using a 3.0-mm Gaussian filter. Final in-plane voxel size was 1.65 mm.

### FDG-PET/CT analysis

Two experienced specialists, one in nuclear medicine and one in radiology interpreted the PET and fused PET/CT images on a *syngo*.via workstation (Siemens Healthineers). The clinical interim PET scans were reported for clinical purposes as usual. All interim PET images were assessed visually using the Deauville five-point scale in comparison with the baseline PET. Score 1 indicates no residual FDG uptake, score 2 indicates FDG uptake ≤ mediastinum, score 3 indicates FDG uptake > mediastinum but ≤ liver, score 4 indicates FDG uptake moderately > liver, score 5 indicates FDG uptake markedly > liver and/or progression of the lesions (Meignan et al., 2009). All pathological lymph nodes and sites of extranodal involvement were evaluated. Additionally, the mean standardized uptake value (SUV_mean_) and standard deviation in spherical volumes of interests placed in the liver were recorded, and the coefficient of variance (COV) was calculated as a characterization of image noise. Images were evaluated unblinded to acquisition time.

Finally, an experienced specialist in nuclear medicine rated image quality based on a subjective perception of image noise and lesion detectability. Image quality was rated on a 3-point scale as follows: good (optimal for clinical interpretation), moderate (adequate for clinical interpretation), or poor (inadequate for clinical interpretation). All images were assessed in one session.

### Estimation of savings when reducing scan time in FDG-PET/CT

Our analysis is based on an FDG-PET/CT scanner performing 2500 whole-body examinations per year, equivalent to approximately ten examinations per day with the scanner operating only on weekdays. In our institution, a standard whole-body FDG-PET/CT examination consists of six-bed positions with an acquisition time of 120 s/bp resulting in a total scan time of 12 min. Two technicians are required to operate a PET/CT scanner.

For cost-saving models, we assume a reduction in whole-body FDG-PET/CT can be achieved without compromising diagnostic quality. Hence, we assume a reduction in acquisition time of 30 s/bp or 60 s/bp.

All the assumptions are listed in Table 1.

**Table 1:** List of assumptions used to calculate estimated savings when reducing scan time of FDG-PET/CT

Assumption	Model value	Explanation
Number of whole-body FDG-PET/CT examinations per year	2500	Equivalent to ten examinations per day when the scanner only operates on weekdays.
Number of bed positions per examination	6	Average number of bed positions in a whole-body FDG-PET/CT examination
Reduction in acquisition time per bed position	30 or 60 s	Assumed reduction in acquisition time in whole-body FDG-PET/CT without compromising image quality
Hourly rate of technicians operating scanner	32 € × 2 = 64 €	It requires two technicians to operate each scanner

### Statistical analysis

Continuous patient parameters are presented as mean ± standard deviation and range. The DS of images made with short scan protocol was compared to images made with normal scan protocol using a two-tailed paired *t* test. COV values were compared to the clinical reference of 15% using one-sample *t* test. A *p* value < 0.05 was considered statistically significant. All statistical analyses were performed using STATA v15.1 SE (StataCorp LP, College Station, Texas).

## Results

### Patients

A total of 20 patients diagnosed with malignant lymphoma were enrolled in this study. Sixty-five percent were female (7 males, 13 females), and mean age was 49 years (range, 20-84 years). Diagnoses included HL (*n* = 14), diffuse large B cell lymphoma (DLBCL) (*n* = 4), and follicular lymphoma (FL) (*n* = 2). Patients received an ^18^F-FDG dose of 4 MBq/kg body weight. Mean body weight was 72 kg (range, 50-113 kg), and mean body mass index (BMI) was 23 (range, 16.5-32.3). The clinical characteristics of patients are shown in Table 2.

**Table 2:** Clinical characteristics of included patients

Characteristics	Number of patients
Total number of patients	20
Male	7
Female	13
Mean age ± standard deviation	49.4 years ± 19.1
Age range	20-84 years
Mean weight ± standard deviation	71.6 kg ± 18.6
Weight range	50-113 kg
Mean BMI ± standard deviation	23.2 ± 4.2
BMI range	16.5-32.3
Diagnosis	
Hodgkin lymphoma	14
Non-Hodgkin lymphoma	6
Diffuse large B cell lymphoma	4
Follicular lymphoma	2

### Deauville score

DS for the 20 patients to different scan times are shown in Table 3. Discordance in DS occurred in one case (DS 3 to DS 2) when comparing 120 s/bp images with 60 s/bp images, and in two cases (DS 3 to DS 2) when comparing 120 s/bp images with 45 s/bp images and 120 s/bp images with 30 s/bp images. Mean absolute differences including standard deviations of DS between images acquired with standard clinical acquisition time (120 s/bp) and shorter acquisition times (90, 60, 45, and 30 s/bp) are listed in Table 4. There was no significant difference in DS between images acquired with shorter scan times (30 s/bp, 45 s/bp, 60 s/bp, 90 s/bp) and images acquired with clinical protocol (120 s/bp).

**Table 3:** The assigned Deauville scores to the different patients at various acquisition times including location of residual uptake for each patient

Patient no.	Location of residual uptake	120 s/bp	90 s/bp	60 s/bp	45 s/bp	30 s/bp
1	Retroperitoneum	3	3	2	2	2
2	Left lower neck	3	3	3	3	3
3	Retroperitoneum	4	4	4	4	4
4	Mediastinum	3	3	3	2	2
5	Left lower neck	4	4	4	4	4
6	Mediastinum	3	3	3	3	3
7	Mediastinum	2	2	2	2	2
8	Mediastinum	3	3	3	3	3
9	Retroperitoneum	5	5	5	5	5
10	Abdomen	3	3	3	3	3
11	Left lower neck	2	2	2	2	2
12	Mediastinum	5	5	5	5	5
13	Mediastinum	2	2	2	2	2
14	Abdomen	4	4	4	4	4
15	Retroperitoneum	3	3	3	3	3
16	Right neck	4	4	4	4	4
17	Left hilum	5	5	5	5	5
18	Mediastinum	5	5	5	5	5
19	Mediastinum	2	2	2	2	2
20	Mediastinum	2	2	2	2	2

**Table 4:** Mean absolute differences ± standard deviation of Deauville score between shorter acquisition times and the reference acquisition time of 120 s/bp

	Mean absolute difference ± standard deviation	*p* value
120-90 s/bp	0	
120-60 s/bp	0.05 ± 0.22	0.330
120-45 s/bp	0.10 ± 0.31	0.163
120-30 s/bp	0.10 ± 0.31	0.163

### Image quality

**Fig. 1:**
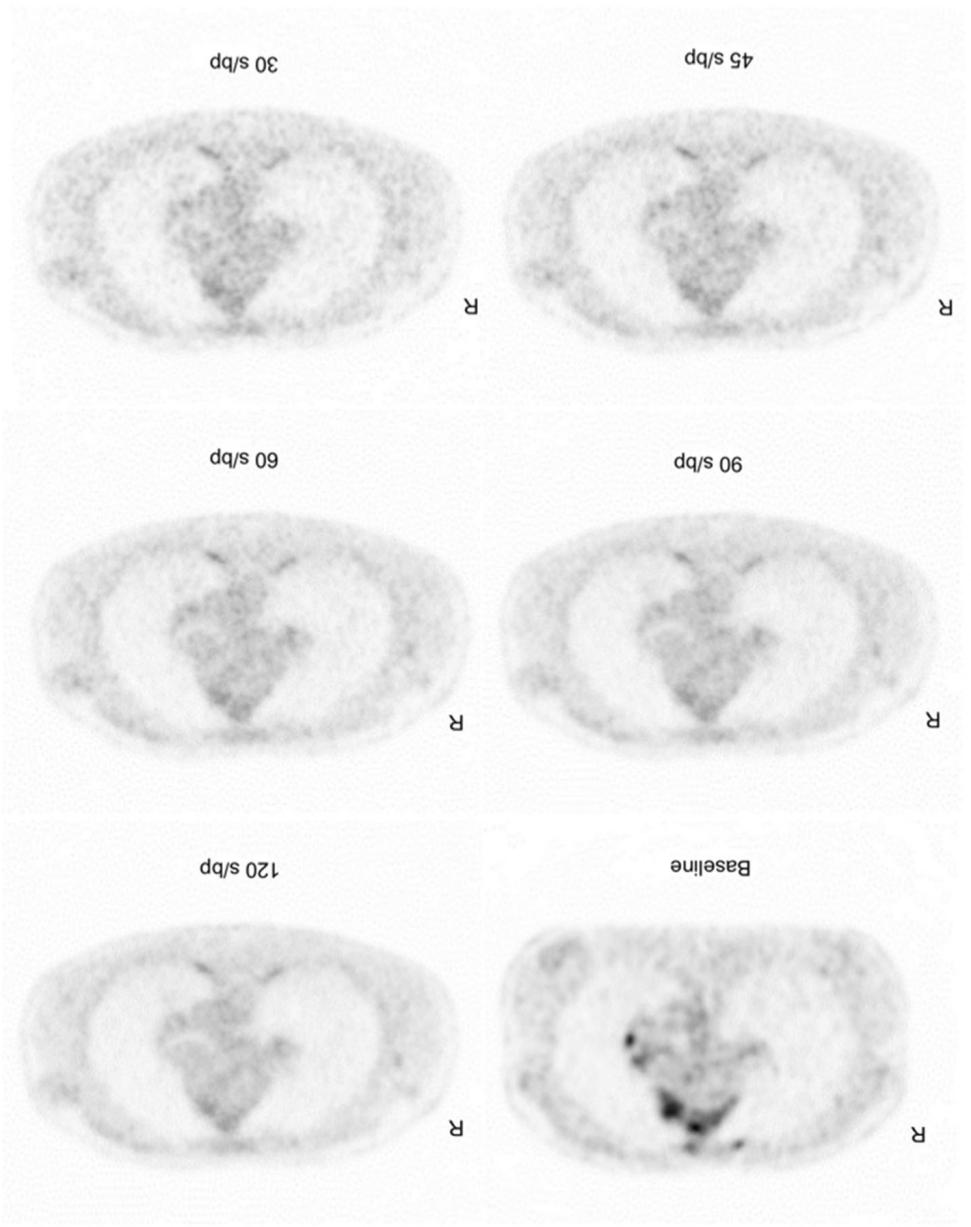
Examples of PET images acquired with different acquisition times. Illustration of image quality of PET images with acquisition times of 120 s/bp, 90 s/bp, 60 s/bp, 45 s/bp, and 30 s/bp. Baseline scan is included to show the location of initial tumor. PET, positron emission tomography; s/bp, seconds per bed position

Table 5 shows the image quality scores versus acquisition time. Image quality was consistently scored good for images acquired with 120 s/bp (100%) and 90 s/bp (100%). Of the 60 s/bp images, 20% were scored good, and 80% were scored moderate. For images acquired with 45 s/bp, 85% were scored moderate and 15% were scored poor. All 30 s/bp images were scored poor (100%). Image quality of PET images with various acquisition times is illustrated in Fig. 1.

**Table 5:** Summary of image quality scores for all 20 patients

	120 s/bp	90 s/bp	60 s/bp	45 s/bp	30 s/bp
**Good**	20 (100%)	20 (100%)	4 (20%)	0	0
**Moderate**	0	0	16 (80%)	17 (85%)	0
**Poor**	0	0	0	3 (15%)	20 (100%)

### COV

**Fig. 2:**
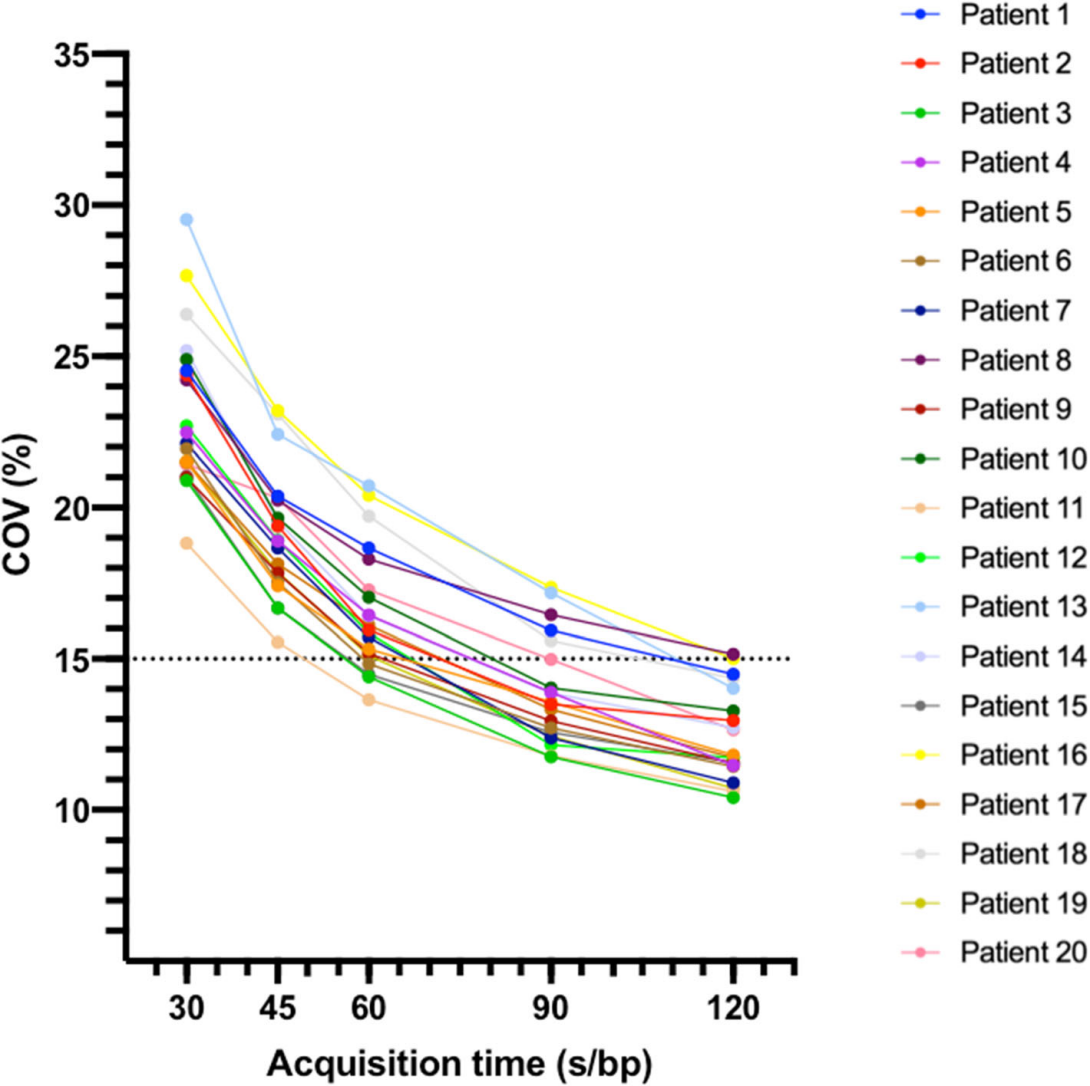
COV according to acquisition time. COV versus acquisition time for each patient. The dotted line indicates the acceptable limit for image noise for clinical image interpretation. COV, coefficient of variance; s/bp, seconds per bed position

Figure 2 shows COV values of the 20 patients versus acquisition time. COV decreases as expected with increasing acquisition time. COV values for images acquired with 120 s/bp and 90 s/bp were found to be significantly less than 15%. COV < 15% is considered as an acceptable noise level for clinical image interpretation (Boellard et al., 2013).

### Estimated savings when reducing scan time in FDG-PET/CT

Based on our analysis, the estimated savings in time and costs are presented in Table 6. Reducing acquisition time to 90 s/bp in whole-body PET/CT corresponds to an estimate of 125 h saved per scanner per year. Consequently, a reduction to 60 s/bp in acquisition time equals to approximately 250 h saved per scanner per year. With a reduction in acquisition time to 90 s/bp and 60 s/bp, the estimated savings in payments to operating technicians are approximately 8000 € and 16,000 € per scanner per year, respectively.

**Table 6:** Estimated savings in time and payment to technicians per scanner per year

	30 s reduction	60 s reduction
Total saved time	125 h	250 h
Saved costs	8000 €	16,000 €

## Discussion

To the best of our knowledge, this is the first study to assess the effect of reducing scan time of response assessment FDG-PET/CT imaging on DS in patients with malignant lymphoma. In this prospective study of 20 consecutive HL and NHL patients, we demonstrate that (1) it is possible to reduce acquisition time of response assessment FDG-PET/CT in lymphoma patients with no significant changes in DS compared to images acquired according to clinical protocol and with no impact on the diagnostic quality of the images. (2) Acquisition time can be reduced without exceeding acceptable noise level for clinical image interpretation. (3) As it is possible to reduce scan times, it is possible to reduce costs.

We found that acquisition time can be reduced to 90 s/bp with no change in DS or image quality compared to images acquired according to clinical protocol. However, from a statistical point of view, we cannot exclude the possibility of minor differences in diagnostic quality between images acquired with 120 s/bp and 90 s/bp. In a larger study sample with a higher level of power, this possible difference might had been observed. Images acquired with 60 s/bp, 45 s/bp, and 30 s/bp did not differ statistically significant in DS from the 120 s/bp images. Additionally, the clinician rated the 60 s/bp images adequate for clinical interpretation.

However, when reducing acquisition time, the image noise increases as shown in Fig. 1. Image noise degrades the image quality and makes the interpretation more difficult for the clinician. For images acquired with shorter acquisition times, it becomes more difficult for the clinician to separate new lesions or small lesions from image noise impairing diagnostic quality. In spite of 6 out of 20 exams had COV ≥ 15%, the diagnostic quality of all images acquired with 90 s/bp were rated good and equivalent to the 120 s/bp images. For images acquired with 60 s/bp, 16 out of 20 exams had a COV ≥ 15%, yet DS did not differ significantly and the clinician rated the images of moderate quality for diagnostic interpretation. This indicates the possibility to reduce scan time even further to 60 s/bp without compromising DS.

In a similar study by Sluis et al., they evaluated the effect of scan time on quantitative and subjective imaging parameters in FDG-PET/CT imaging performed on 30 patients diagnosed with non-small cell lung carcinoma, esophageal cancer, or lymphoma (van Sluis et al., 2019). Images were acquired according to EANM guidelines using a Biograph Vision PET/CT and reconstructed using the clinically vendor recommended protocol as we did in the present study. They found it was possible to reduce the scan time of FDG-PET/CT from 180 s/bp to 60 s/bp with no significant changes in quantitative parameters. In addition, they found a significant increase in image noise with shorter scan times similar to our findings. Thus, suggesting that a reduction in scan time may be feasible when using more advanced PET/CT scanners. Another study by Sonni et al. assessed the impact of various acquisition times on image quality using a silicon photomultipliers-based PET/CT scanner in 58 cancer patients (Sonni et al., 2018). They found it was possible to acquire images at 90 s/bp using the Discovery Meaningful Insights PET/CT without compromising the image quality, which agrees well with our findings.

The implementation of new technologies such as PSF and TOF in new generation PET/CT scanners potentially allows for shorter scan times and/or reduction in administered tracer dose. PSF and TOF have been found to improve visual detection of small lesions but is also found to increase SUVs in small lesions while SUVs in reference organs such as the liver is not affected (Andersen et al., 2013; Akamatsu et al., 2014; Kuhnert et al., 2016). Specifically, SUV_max_ has been found unreliable for subcentimeter lesions when using PSF reconstruction (Munk et al., 2017). Consequently, tumor-to-liver ratio, which is used in Deauville criteria to discriminate between a positive and negative exam in HL and NHL patients, has been shown to be PET system and image reconstruction method dependent. This is of great importance to response evaluation as the effect may compromise the DS, and thereby affect the choice of medical therapy.

A recent study by Ly et al. investigated whether using a new generation PET/CT scanner with block-sequential regularization expectation-maximization algorithm reconstruction, which includes PSF, would influence DS compared with reconstructions following current EANM guidelines in 57 patients diagnosed with malignant lymphoma (Ly et al., 2019). They found that the choice of reconstruction method has a significant impact on DS and therapy response evaluation. This confirms the need for an updated version of the Deauville criteria to accommodate new generation PET/CT technologies as Deauville criteria is derived from studies performed in previous generation PET/CT systems with the use of non-TOF and non-PSF reconstruction.

Despite a monocentric setup and limited number of patients, we have demonstrated the impact of reducing scan time in FDG-PET/CT on DS in this prospective study, in which we only included patients with a histological verified HL, DLCBL, or FL diagnosis referred to response assessment FDG-PET/CT. We followed clinical guidelines and assessed interim PET images according to gold-standard Deauville criteria. Our findings are highly in the interest of the patients, as a shorter scan time would potentially increase the comfort and compliance of the patients. Considering the ALARA principle, it could be relevant to convert a shorter scan time to a reduced injected activity to the patient. This would result in a decreased radiation exposure for patients as well as for the medical staff. It would be of interest to validate our findings and to investigate the potential to reduce injected activity in a clinical setting.

With the shorter scan time, patient throughput could be increased with increased cost-effectiveness. Our simple model shows that it is financially beneficial to reduce acquisition time to 90 s/bp or 60 s/bp in whole-body FDG-PET/CT with estimated savings of 8000 or 16,000 € per scanner per year, respectively. We recognize our analysis was conducted with a simplified model not taking the work around with patient preparation into account, we only calculated pure in-scanner time. We are aware of the fact that acquisition time on the new generation scanners is one of the smallest parts of a PET/CT scan. The assumption of a PET/CT scanner performing 2500 response assessment FDG-PET/CT examinations per year is a theoretical example. A case mix of only malignant lymphoma patients is highly unlikely as a PET/CT scanner performs numerous types of examinations in different patient groups using different tracers during daily clinical routine. In addition, the assumed values are based on our clinic in Denmark and must be adjusted for specificity when analyzing other countries.

Finally, the use of artificial intelligence in medical imaging should be mentioned. Through the use of artificial intelligence on low-dose PET images, image noise can be significantly reduced to produce high-quality PET images similar to full-dose PET images adequate for clinical interpretation (Chen et al., 2019). The application of artificial intelligence could potentially lead to further reductions in scan time and/or injected radiotracer without compromising diagnostic image quality.

## Conclusion

We have shown that it is possible to reduce the acquisition time from 120 s/bp to  90 s/bp of response assessment FDG-PET/CT imaging in patients diagnosed with HL, DLBCL, and FL without compromising Deauville score or image quality. This is of great importance for the comfort of the patients and for the economy.

## Data Availability

Not applicable.
